# Reactive gliosis and neuroinflammation: prime suspects in the pathophysiology of post-acute neuroCOVID-19 syndrome

**DOI:** 10.3389/fneur.2023.1221266

**Published:** 2023-08-24

**Authors:** Jacob Saucier, Dominique Comeau, Gilles A. Robichaud, Ludivine Chamard-Witkowski

**Affiliations:** ^1^Centre de Formation Médicale du Nouveau-Brunswick, Moncton, NB, Canada; ^2^Faculté de Médecine et des Sciences de la Santé, Université de Sherbrooke, Sherbrooke, QC, Canada; ^3^Centre de médecine de précision du Nouveau-Brunswick, Vitality Health Network, Moncton, NB, Canada; ^4^Department of Chemistry and Biochemistry, Université de Moncton, Moncton, NB, Canada; ^5^Atlantic Cancer Research Institute, Moncton, NB, Canada; ^6^Department of Neurology, Dr. Georges-L.-Dumont University Hospital Centre, Vitality Health Network, Moncton, NB, Canada

**Keywords:** post-COVID syndrome, NeuroPASC, reactive gliosis, neuroinflammation, microglial reactivity, reactive astrocytes

## Abstract

**Introduction:**

As the repercussions from the COVID-19 pandemic continue to unfold, an ever-expanding body of evidence suggests that infection also elicits pathophysiological manifestations within the central nervous system (CNS), known as neurological symptoms of post-acute sequelae of COVID infection (NeuroPASC). Although the neurological impairments and repercussions associated with NeuroPASC have been well described in the literature, its etiology remains to be fully characterized.

**Objectives:**

This mini-review explores the current literature that elucidates various mechanisms underlining NeuroPASC, its players, and regulators, leading to persistent neuroinflammation of affected individuals. Specifically, we provide some insights into the various roles played by microglial and astroglial cell reactivity in NeuroPASC and how these cell subsets potentially contribute to neurological impairment in response to the direct or indirect mechanisms of CNS injury.

**Discussion:**

A better understanding of the mechanisms and biomarkers associated with this maladaptive neuroimmune response will thus provide better diagnostic strategies for NeuroPASC and reveal new potential mechanisms for therapeutic intervention. Altogether, the elucidation of NeuroPASC pathogenesis will improve patient outcomes and mitigate the socioeconomic burden of this syndrome.

## Graphical Abstract

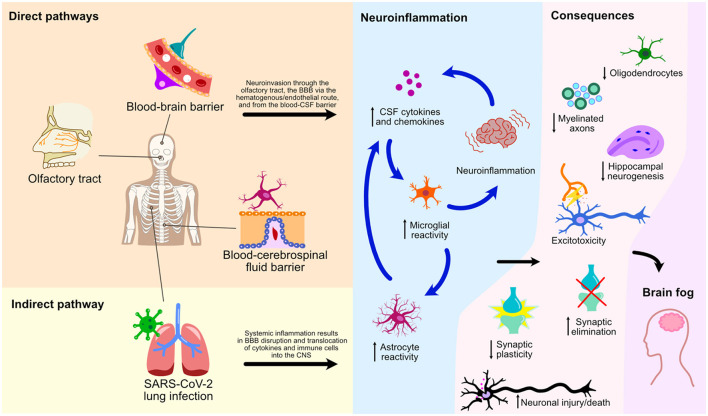

## 1. Introduction

It has been established that the pathophysiology of severe acute respiratory syndrome coronavirus 2 (SARS-CoV-2) entails long-term symptomatic repercussions in infected patients. The Center for Disease Control and Prevention (CDC) defines post-acute sequelae of COVID-19 (PASC) as the persistence of COVID-19 symptoms beyond 4 weeks of the initial infection (1). PASC is a multi-organ disease with a plethora of clinical manifestations including dyspnea, cough, fibrotic changes on pulmonary imaging, palpitations, chest pain, thromboembolic events, chronic kidney injury, fatigue, endocrine disruption, hair loss, and multiple neuropsychiatric manifestations (2).

The long-term impact of COVID-19 on the central nervous system (CNS) has been a growing area of concern, with its consequences referred to as post-acute neurological symptoms of COVID-19 (NeuroPASC). NeuroPASC's most prevalent symptom, cognitive impairment, has been reported in 28.85% of patients following COVID-19 infection according to a recent systematic review and meta-analysis (3). However, upon neuropsychological evaluation, cognitive deficits have been objectified in over 50% of COVID-19 patients (4–8). The term “*brain fog*” has been extensively used in the literature and mainstream media to illustrate the cognitive state of NeuroPASC. It refers to a non-specific constellation of symptoms, including the subjective complaints of poor attention, executive function, and problem solving (9), that may impede daily activities and interpersonal relationships (10). Various conditions may mimic COVID-19's brain fog, including anxiety and mood disorders, traumatic brain injury, chronic fatigue syndrome, and cancer-related cognitive impaired, coined “chemo-fog” (10). Nonetheless, other longstanding neurological symptoms such as fatigue, headache, myalgia, dysautonomia, deficits in verbal fluency, attention loss, executive functions, and memory impairments have been objectified following SARS-CoV-2 infection (6, 9, 11). Recently, cognitive inhibition deficits were reported to be highly prevalent among COVID-19 cases as 38.8% of patients expressed sustained deficits in cognitive inhibition for up to 16 months following COVID-19 infection (8). While other cognitive domains such as cognitive efficiency and executive functions longitudinally improved, cognitive inhibition remained persistently poor over time. An extensive literature has described the psychiatric manifestations of NeuroPASC. Accordingly, a recent meta-analysis has documented the prevalence of long-term neuropsychiatric manifestations following SARS-CoV-2 infection, including sleep disturbances (27.4%), fatigue (24.4%), anxiety (19.1%), and post-traumatic stress disorder (PTSD) (15.7%) (12). Similarly, clinically relevant depressive symptoms in convalescent individuals were estimated between 21 and 45% in COVID-19 patients (13). Efforts to identify risk factors of NeuroPASC development following SARS-CoV-2 infection have produced heterogeneous results across different cohorts (14). While cognitive impairment was greater in ICU compared to non-ICU patients in some studies (8, 15, 16), other reports did not observe any differences in cognitive impairment in the function of infection severity (7, 17). Nonetheless, consistent findings across studies identified female sex (18, 19), older age (19, 20), and previous dementia or cognitive complaints (19, 21) as risk factors for NeuroPASC development.

Several theories have been proposed to explain these neurocognitive symptoms, including inflammatory changes, hypoxia, coagulopathy, vascular endothelial, dysfunction and direct viral invasion of the neurological tissue (22). Although the precise mechanisms remain elusive, six mechanisms have been proposed: i) systemic immune response-mediated neural dysregulation; ii) direct CNS invasion; iii) auto-immune responses; iv) latent pathogen reactivation; v) cerebrovascular thrombosis; and vi) multi-organ dysfunction (23). In this mini-review, we have highlighted the leading hypotheses and pathological mechanisms supporting NeuroPASC, through the consequential disturbance of reactive microglia and astroglia, which lead to persistent neurocognitive symptoms of PASC.

### 1.1. Glial cell reactivity

**Figure 1:**
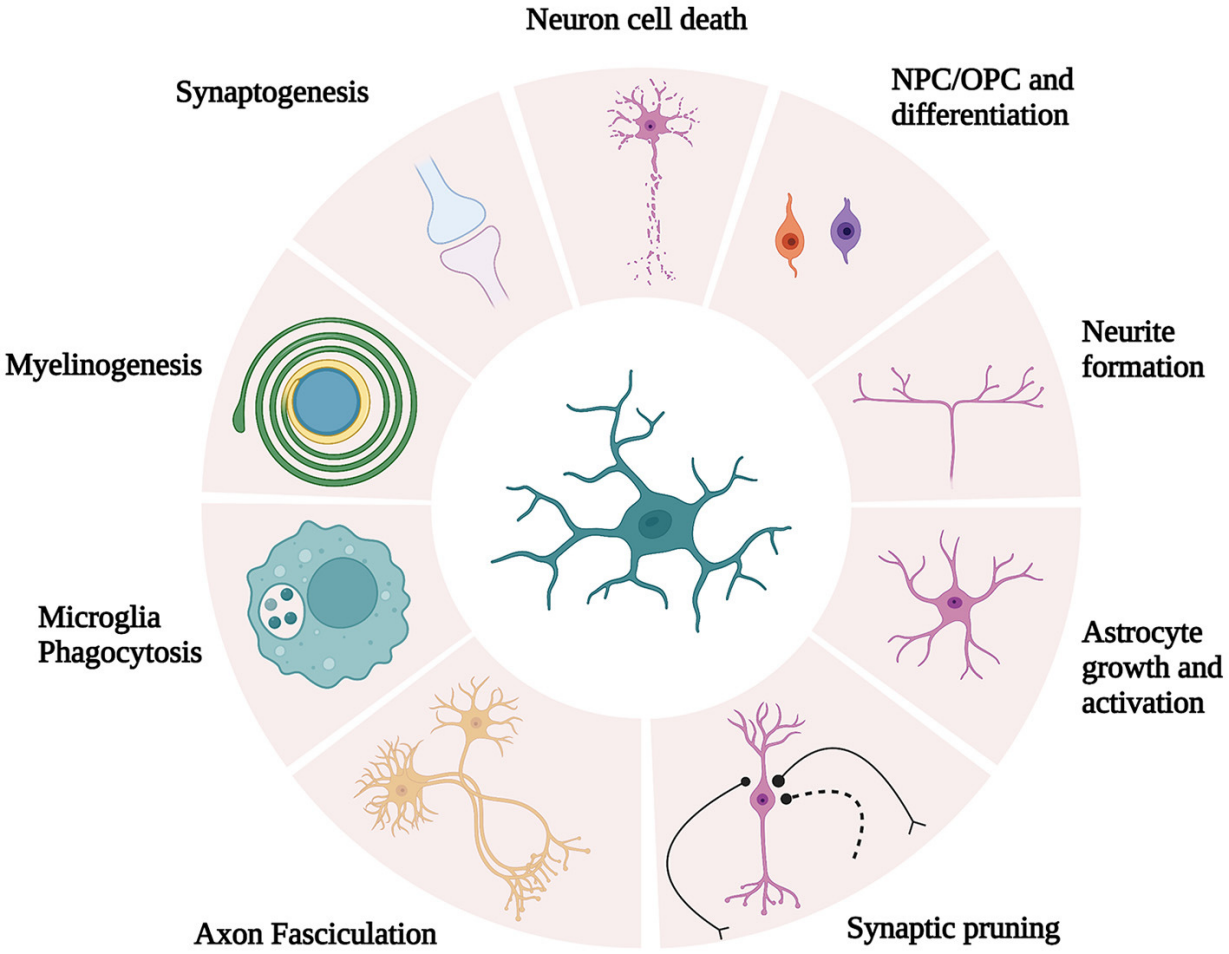
Schematic illustration of microglia function. Microglia are responsible for crucial functions in the CNS, including synaptic pruning and synaptogenesis, axon fasciculation and neurite formation, programmed cell death, astrocyte activation and proliferation, and oligodendrocyte differentiation and myelogenesis. [Modified and reproduced from Wright-Jin and Gutmann (27), Microglia as Dynamic Cellular Mediators of Brain Function, with the permission of Cell Press.].

Maintenance of optimal cognitive function is a complex process that requires coordination between neuron function and glial cells (24). In recent years, significant interest has been allocated to glial cell (i.e., microglia, astrocytes, and oligodendrocytes) dysfunction during cognitive impairment. In fact, the dysregulation of glial cell function leads to cognitive impairment associated with numerous neuropathologies, including metabolic syndromes (24) and neurodegenerative diseases such as Parkinson's (25) and Alzheimer's (26) diseases. Microglia, the resident immune phagocytes of the CNS, are essential for learning, memory, and behavior regulation in the adult brain (27). In addition to immune surveillance and phagocytosis, microglia are also responsible for other crucial functions in the CNS, including synaptic pruning and synaptogenesis, axon fasciculation and neurite formation, programmed cell death, astrocyte activation and proliferation, and oligodendrocyte differentiation and myelogenesis (27) (Figure 1). Based on the concept of cellular polarization, cells were separated into two phenotypically distinct sub-populations characterized by opposing effects on the CNS. Specifically, the classical (M1) microglial subset was believed to be responsible to produce pro-inflammatory mediators, which induced inflammation and neurotoxicity. Conversely, M2 was assumed to release anti-inflammatory factors, which confer neuroprotectivity. With the advent of technology, M1 and M2 microglia are portrayed as brute oversimplifications to illustrate antagonistic states in both healthy and diseased brains (28). Microglia are likely to be significantly more complex as microglial subset identity and function are intricately regulated by microglial metabolic states and the environmental profiles of signaling mediators (e.g., cytokines and neurotransmitters) (24).

Complex microglial–astrocyte interactions also form a delicate equilibrium in CNS health. Indeed, cellular dysfunction from either cell population or the maladaptive synergistic interactions between microglia and astrocytes can result in neurotoxicity and alter synaptic plasticity through numerous mechanisms (29, 30). With a crucial role in brain homeostasis, astrocytes regulate CNS blood flow, glucose metabolism, and the recycling of neurotransmitters (24). Astrocytes are also depicted as master regulators of synaptic activity by controlling synaptic junction plasticity and mediating synapse elimination to avoid excitotoxicity (31, 32). Reminiscent of microglia's obsolete nomenclature, astrocytes are classified into two distinct sub-populations (A1 and A2) based on their reactivity and function (30). On the one hand, A1 reactive astrocytes produce pro-inflammatory soluble mediators, which are mainly induced by the NF-κB signaling cascade (33). On the other hand, A2 reactive astrocytes generate anti-inflammatory mediators and many neurotrophic factors induced by STAT3 activation. As a result, reactive A1 astrocytes provoke neurotoxicity and neuronal death, whereas A2 astrocytes promote survival and neuron growth (33).

Upon cerebral insult, astrocytes undergo drastic phenotype change referred to as reactive astrocytosis, induced due to an upregulation of pro-inflammatory cytokines by neuroinflammatory microglia such as (interleukin) IL-1α, IL-1β, tumor necrosis factor-alpha (TNF-α), and the complement component 1q (C1q) (29, 30, 33). As a result, neurotoxic A1 reactive astrocytes display decreased function in synaptic formation and phagocytic capability. Furthermore, A1 reactive astrocytes promote significant neurotoxicity, which leads to cell death of cortical neurons and mature differentiated oligodendrocytes (30, 34). Moreover, inflammatory microglia further accentuate NF-κB signaling, leading to A1 astrocyte population remodeling and neurodegeneration (35). A study by Saggu et al. (36) has shown that astroglial-mediated NF-κB activation is associated with white matter damage and cognitive impairments in vascular dementia models (36). While microglial activation alone is insufficient to initiate cell death in the CNS, microglial activation potentially enhances neurological damage by inducing reactive astrocytosis, resulting in neurodegeneration (30).

As for oligodendrocytes, they are responsible for axonal myelination, which regulates action potential conduction velocity, essential for neural circuit dynamics (37). Oligodendrocytes are also important contributors to neurodegenerative diseases including Alzheimer's disease, amyotrophic lateral sclerosis, and multiple system atrophy. More recently, studies have shown that, in addition to myelination, oligodendrocytes are required for the integrity and survival of axons independent of myelin itself (38). Mechanistically, oligodendrocytes foster glycolytic metabolism, which provides axons with energy-rich metabolites.

Altogether, the coordinated signaling between microglia, astrocytes, and oligodendrocytes is essential for homeostasis and CNS health.

## 2. SARS-CoV-2-mediated activation of glial cells

### 2.1. Indirect pathway: peripheral immune cell activation and CNS infiltration

Acute and chronic CNS inflammation alike have drastic repercussions on glial circuitry and cytokine expression profiles, which result in dysfunctional immune signaling and synaptic plasticity (39). As a result of the intricate equilibrium that composes glial cell homeostasis, various neuroinflammatory states including chemotherapy (40) and notably COVID-19 infection (41), disrupts glial lineage, pertaining to glial population proliferation, differentiation, and maturation. Following COVID-19 infection, an upregulation of pro-inflammatory chemokine-enhanced microglial populations and an impairment of oligodendrogenesis in mice models led to neurological disturbance in the absence of direct viral invasion (41).

Neuroinflammation underlies one of the leading theories to explain CNS injury during SARS-CoV-2 infection and is a consequence of the well-documented systemic cytokine storm and subsequent increase in blood–brain barrier (BBB) permeability (14, 42). Through its spike surface glycoprotein, SARS-CoV-2 enters the host cells by binding to its angiotensin-2 converting enzyme (ACE-2) receptors, which consequently initiates an important inflammatory response (13, 43). Brain–blood barrier disruption from systemic inflammation facilitates neuroinflammation through neural invasion of inflammatory cytokines, which further stimulates cytokine secretion from the microglia (42). Accordingly, a study in rats has shown that exposure to a partial subunit of the SARS-CoV-2 spike protein (i.e., S1 protein subunit) elicits innate immune response through a pathogen-associated molecular pattern (PAMP), which triggers microglial activation and neuroinflammation in the absence of active virions (44). The S1 spike protein also activates the NRLP3 inflammasome that plays a pivotal role in innate immunity and inflammatory signaling triggered by PAMPs (45). This pathway leads to NF-κB activation, pro-inflammatory cytokine production (i.e., IL-1β and IL-18), and subsequent glial reactivity, all of which are associated with neurodegenerative diseases (46). Meanwhile, microglial activation *via* NF-κB signaling induces reactive astrocytosis, which in turn leads to excitotoxicity, white matter damage, and loss of myelin plasticity, in addition to oligodendrocyte and neuronal cell death (14, 35, 44).

Neuroinflammatory pathways that alter CNS homeostasis are linked to cognitive and neuropsychiatric complications (43). The systemic immune-inflammation index, which reflects the immune response and systemic inflammation based on a ratio of peripheral lymphocyte, neutrophil, and platelet counts (SII = platelets × neutrophils/lymphocytes), has been found to predict depressive symptomatology and cognitive dysfunction 3 months following initial infection (47). Even in the absence of direct CNS viral infiltration, consequential production of peripheral cytokine profiles associated with the host's antiviral response may be sufficient to induce neuroinflammatory reactions and/or compromise the integrity of the blood–brain interface. As a result, peripheral immune cells migrate through the BBB into the CNS and induce microglia-derived cytokines, which interfere with neurotransmission (14, 42). These mechanisms have mostly been established using experimental models. For example, mild respiratory illness in AAV-hACE2 mice (48) following intranasal delivery of SARS-CoV-2 was sufficient to induce potent microglial reactivity in the sub-cortical white matter upon pathological examination of the mice brain tissue (41). Moreover, Klein et al. (49) compared the hamster models of SARS-CoV-2 to pathological specimens of human patients deceased from COVID-19, demonstrating similar pathological changes in the absence of viral neuroinvasion. These changes included abnormal BBB permeability, microglial activation, loss of hippocampal neurogenesis, and expression of IL-1β and IL-6 within sub-cortical structures (49).

Neuroinflammation during acute SARS-CoV-2 infection may consequently induce brain parenchyma and vessel alterations that further foster the inflammation of neurons and supportive cells (14). Additionally, such neuroinflammation could be a catalyst for microvascular thrombosis and ischemic brain injury during the COVID-19 infection (50). Magnetic resonance imaging (MRI) from a deceased COVID-19 patient revealed volumetric and micro-structural brain abnormalities, which were accompanied by several neuropathological lesions reminiscent of vascular and demyelinating etiology (51). Any combination of these events could lead to BBB disruption and subsequent immune cell infiltration of the CNS causing microglial activation and neuroinflammation in the absence of direct viral invasion of the CNS.

### 2.2. Direct pathways

Glial activation and neurotoxicity may result from the direct routes of SARS-CoV-2 infection. In a study where transgenic mice models expressing recombinant human ACE-2 were infected with SARS-CoV-2, investigators found viral particle (spike protein) infiltration within the CNS and an abundance of activated microglia in the proximity of the infected tissue (46). The utilization of human monocyte-derived microglia infected with SARS-CoV-2 revealed that viruses enter these cells through ACE-2 receptor binding in the absence of viral replication. More interestingly, they observed that the infected cells induced NLRP3 inflammasome activation and a potent pro-inflammatory response accompanied by IL-1β overexpression (46). Mechanistically, these neuroinflammatory events were shown to be NF-κB dependent as the utilization of NF-κB inhibitors led to complete inhibition of Il-1β release. Another study conducted by Samudyata et al. (52) established a brain organoid model with innately developing microglia (52). Such *in vitro* invasion assays on microglial cells co-cultured with SARS-CoV-2 demonstrate the loss of post-synaptic termini and neuronal cell death. Transcriptomic profiling of microglia exposed to SARS-CoV-2 revealed gene expression signatures that closely resembled neurodegenerative disorders (52). Nevertheless, it is worth noting that SARS-CoV-2 antigens and RNA have rarely been detected in the CSF of COVID-19 patients (53, 54) while only detected in a minority of human brain autopsies (55). Heterogenous study results have resulted in controversy surrounding the neuroinvasive properties of SARS-CoV-2. This section will explore pathways by which CNS infiltration of SARS-CoV-2 of viral proteins may result in microglial activation and neuroinflammation during COVID-19 infection.

#### 2.2.1. Olfactory route

The presence of ACE-2 receptors along the olfactory tract suggests that the neurological manifestations of COVID-19 could be caused by direct neurological infiltration *via* the olfactory route (56, 57), a common entry site to several other respiratory viruses (58). CNS viral dissemination to the amygdala, hippocampus, and entorhinal cortex could then be possible through the connecting olfactory bulb, where SARS-COV-2 RNA has been found in approximately 20% of post-mortem brains from deceased COVID-19 patients (59). Numerous imaging studies also support this hypothesis (59–61). For example, neuroimaging from a cohort of 785 participants (including 401 participants scanned before and after COVID-19 infection) discovered significant longitudinal effects in SARS-CoV-2 cases including a decrease of thickness and tissue contrast from the orbitofrontal cortex and the parahippocampal gyrus gray matter, changes in tissue damage markers in olfactory cortex-related regions, and a global reduction in brain volume (61). Previously infected individuals from the latter cohort also demonstrated cognitive decline post-infection. Together, imaging data originating mainly from the limbic system could highlight COVID-19-mediated neurodegeneration through the olfactory pathways, neuroinflammatory events, and loss of sensory input caused by anosmia (61). Other imaging studies in COVID-19 patients using MRI cerebral imaging have enabled researchers to observe an increase in olfactory bulb signal intensity and volume size (60). Positron emission tomography (PET) has also shown reduced 18-fludeoxyglucose of orbitofrontal hypometabolism in patients with anosmia (62). Altogether, these findings suggest a role for imaging technologies in the detection and progression of direct neurological infiltration and pathogenesis of COVID-19 infection through the olfactory tract.

#### 2.2.2. Hematogenous spread and endothelial pathology

Perturbation of BBB permeability has been well documented during the infection of various respiratory viruses (63). Of note, cerebral endothelial cells, which comprise the BBB, are prone to SARS-CoV-2 infection through cell surface expression of receptors NRP1, BSG, and low levels of ACE-2 (64). Furthermore, SARS-CoV-2 has been shown to cross the BBB by transcellular pathways, accompanied by basement membrane disruption in mice models (65). As a result, vascular permeability increases and leads to perivascular cell infiltration and neuronal cell death. Wenzel et al. (64) have demonstrated brain endothelial cells infection; the expression of SARS-CoV-2 main protease (Mpro) cleaves the host protein NF-κB essential modulator (NEMO), which is an essential modulator of NF-κB-mediated survival (64). By ablating NEMO, M^pro^ induces microvascular pathology, BBB disruption, endothelial cell death, and neuroinflammation. Similarly, ACE-2 (66) and NRP1 (67) receptors can be found in astrocytes, which are in direct contiguity with the BBB. Astrocyte infection by SARS-CoV-2 is further supported by the detection of the S1 spike gene transcripts and protein in the cerebral vasculature of COVID-19 patients (64) and the description of S1 spike-positive astrocyte in post-mortem human samples (67). Subsequently, *in vitro* neural stem cell-derived human astrocytes were exposed to SARS-CoV-2, resulting in astrocyte infection through spike-NRP1 interactions (67). The resulting astrocyte phenotype decreased neuronal viability while promoting neuronal apoptosis (67).

Previous studies have also demonstrated the occurrence of neuropathological events mediated by the S1 protein of SARS-CoV-2. Accordingly, SARS-CoV-2 virions are known to spontaneously shed S1 protein subunits, which can be found in the plasma of COVID-19 patients (44, 68). This pro-inflammatory protein has also been found in human cerebral endothelial cells upon autopsy in the absence of viral RNA and is strongly co-localized with inflammatory mediators including caspase-3, TNF-α and IL-6 (69). In mouse models, S1 spike protein injection leads to endothelial cell damage with increased expression of TNF-α and IL-6, which co-localized with the S1 spike subunit (69). Similarly, non-primate models have demonstrated the presence of SARS-CoV-2 nucleocapsid protein in endothelial cells of the cerebral vasculature (70). Altogether, the expression of SARS-CoV-2 compatible receptors in cerebral structures, in addition to the discovery of SARS-CoV-2 genetic material and viral proteins in the endothelial tissue and astrocytes, suggests that viral invasion or viral protein infiltration of cerebral vasculature could be a mechanism that leads to microglial activation and neuroinflammation.

#### 2.2.3. Cerebrospinal fluid

Another proposed route for SARS-CoV-2 infection is through the cerebrospinal fluid (CSF). In a study utilizing human-pluripotent-stem-cell-derived brain organoids to examine SARS-CoV-2 neurotropism, ACE-2 positive choroid plexus epithelial cells were amenable to infection, which leads to an initial disruption of the blood–CSF barrier followed by a subsequent complete breakdown of barrier integrity (71). Infection of these organoids has been associated with transcriptional dysregulation and cell death, suggestive of a neuroinflammatory response and deficits in cellular functions (72). Although some studies have shown SARS-CoV-2 PCR positivity in patient's CSF samples, other studies have contradicted this notion (73). While the neuroinvasive properties of SARS-CoV-2 through the blood–CSF barrier have not been confirmed, a more likely mechanism involves barrier leakage, leading to the translocation of immune cells and cytokines that sustain neuroinflammation (71).

## 3. Reactive gliosis as a culprit of NeuroPASC

### 3.1. Current evidence of microglial reactivity in NeuroPASC

A recent study on AAV-hACE2 mice models with mild SARS-CoV-2 respiratory infection has demonstrated a prominent increase in pro-inflammatory cytokine and chemokine profiles (e.g., IFN-γ, IL-6, TNF-α, CXCL10, CCL7, CCL2, and CCL11) in the CSF and serum samples as rapid as 7 days post-infection (41). Longitudinal evaluation of pro-inflammatory mediators revealed that while serum levels of these mediators normalized after 7 weeks, there was a progressive increase of CSF cytokines/chemokines levels over time. Notably, CCL11, a cytokine associated with cognitive impairment (74), remained persistently elevated in the CSF over time, suggesting that isolated respiratory infection with SARS-CoV-2 can result in prolonged changes in CSF cytokine profiles, leading to persistent neuroinflammation (41). The latter study has also demonstrated that mice infected with SARS-CoV-2 displayed white matter microglial reactivity for at least 7 weeks, which culminated in oligodendrocyte death, axonal demyelination, and impaired mechanisms of cellular homeostasis and neuron generation in the hippocampus. These findings align with recent studies highlighting BBB disruption, microglial activation, aberrant cytokine expression, and suppression of hippocampal neurogenesis in brain samples from post-mortem COVID-19 patients (49). Moreover, Schultheiß et al. (75) demonstrated elevated serum cytokine profiles up to 8 months post-infection in a cohort of COVID-19 patients manifesting mostly mild-to-moderate infection severity (75). Interestingly, persistently elevated levels of serum IL-1β, IL-6, and TNF-α correlated with PASC symptoms of dyspnea, fatigue, and cognitive impairment. Further examination also suggested that these cytokines were constitutively secreted by resident monocytes/macrophages in the lungs (75). In parallel, a study by Peluso et al. (76) revealed that an increase in plasma IL-6, TNF-α and glial fibrillary acidic protein (GFAP), an axonal structural protein and biomarker of glial cell activation, predicts NeuroPASC symptoms in SARS-CoV-2 infected patients (76).

Cognitive dysfunction is also correlated with increased immunoregulatory pathway protein expression and a downregulation of inflammatory and antiviral response proteins (77). Moreover, individuals with NeuroPASC exhibit deficient systemic humoral immunity response to various SARS-CoV-2 antigens (Spike, S1, S2, RBC, and Nc) when compared to non-PASC COVID-19 control patients. Elevated levels of serum IgG specific to SARS-CoV-2 are associated with improved NeuroPASC clinical outcomes possibly due to enhanced viral clearance (78), while individuals who experience severe neurological injury following acute COVID-19 infection tend to elicit elevated levels of CSF SARS-CoV-2 specific antibodies (79). Distinct T-cell response and effector signatures in addition to unique CSF humoral responses highlight the significance of humoral immunity alterations and pathogenic outcomes of NeuroPASC (77, 78). Taking into consideration that mild respiratory infection and systemic inflammation can lead to BBB permeability disturbances combined with microglial reactivity (41), one could suggest that immunologic alterations (77, 78) and persistent systemic inflammation following COVID-19 (75) may be a catalyst for chronic neuroinflammation and glial reactivity in previously primed microglia.

### 3.2. Microglial priming and persistent neuroinflammation in NeuroPASC

Considering the detrimental role of persistent microglial reactivity in neurodegenerative diseases, such reactive states could also be key to NeuroPASC pathogenesis. Accordingly, a key concept in AD trajectory known as microglial priming is associated with aging and systemic inflammation (80). Fundamentally, microglia priming renders them more susceptible to secondary inflammatory events, which in turn promotes microglial differentiation to pro-inflammatory subtypes and triggers an exaggerated inflammatory response in response to subsequent stimuli (80). This phenomenon may explain why the prevalence of NeuroPASC is higher in older adults (20). Although the specific mechanisms initiating microglial priming remain to be elucidated, it is generally accepted that chronic inflammation and/or repetitive inflammatory stimuli are a governing factor. Recently, Albornoz et al. (46) have demonstrated that the SARS-CoV-2 S1 spike protein acts as an NLRP3 inflammasome and microglial primer, setting the stage for increased reactivity to inflammatory stimuli (46). Persistent glial reactivity and chronic neuroinflammation in neurodegenerative diseases can be attributed to an exaggerated inflammatory response upon repeated exposition to pathological stimuli (80), such as β-amyloid plaques and alpha-synuclein in AD (80) and Parkinson's disease (PD) (81), respectively. Similarly, the persistent systemic inflammation in PASC (75) could represent a stimulus with the capacity to longitudinally promote microglial reactivity, leading to maladaptive neuroinflammation in microglia previously primed during the wake of SARS-CoV-2 infection. Keeping these mechanisms in mind, SARS-CoV-2 infection and pathogenesis could potentially trigger neurodegenerative events reminiscent of AD and PD (82). As such, there exists a positive correlation between COVID-19 infection and its severity with the risk of AD development (83). Moreover, COVID-19 may exacerbate motor and non-motor symptoms in PD patients (84).

There are considerable parallels between SARS-CoV-2 and influenza sequelae. Iosifescu et al. (20) and Taquet et al. (85) compared neurological and psychiatric sequelae following these viral infections. The incidence of long-term COVID-19 and influenza-related neuro-sequelae was 2.58 and 2.06% (20) and 3.01 and 1.83% (85), respectively. The average onset of NeuroPASC symptoms was 138 days following the initial infection vs. 238 days for influenza sequelae (20). The occurrence of altered mental status was significantly greater in NeuroPASC patients (17%), but there were no statistically significant differences in other clinical signs and symptoms when compared to influenza. These symptoms include anxiety, depression, dizziness, fatigue, headaches, nausea, seizures, and strokes (20). From a pathophysiological perspective, respiratory influenza infection elicits neuroinflammation through pro-inflammatory cytokines secretion and microglial reactivity (86, 87). These processes alter BBB permeability, structural hippocampal plasticity, and may underlie cognitive dysfunction (86, 87). Fernández-Castañeda et al. (41) compared CSF pro-inflammatory cytokine profiles at 7 days and 7 weeks post-infection between mice models of SARS-CoV-2 and H1N1 influenza, revealing distinct profiles, with some overlap. Of note, CCL11, a cytokine associated with cognitive impairment (74) remained persistently elevated in both SARS-COV-2 and H1N1 models. A comparison of microglial reactivity revealed similar hippocampal pathology at 7 days and 7 weeks post-infection. However, unlike respiratory COVID, sub-cortical white matter integrity in H1N1 mice was preserved at 7 weeks, with a resolution of acute microglial reactivity and oligodendrocyte loss (41).

Alternatively, microglial activation during acute SARS-CoV-2 infection could be sufficient to induce maladaptive inflammatory pathways, leading to chronic neuroinflammation and NeuroPASC in the absence of longitudinal peripheral stimuli. This phenomenon has been described following traumatic brain injury (TBI) in human brain samples, where densely packed reactive microglia are responsible for chronic neuroinflammation and white matter degradation (88). In fact, persistent inflammatory pathology was observed in over a quarter of TBI cases and for up to 18 years following the initial brain injury (88). Studies also showed that the ensuing microglial activation and neuroinflammation from TBI results in cognitive impairment and predispose to AD (89).

A comparable syndrome is cancer-therapy-related cognitive impairment, commonly referred to as “chemo-fog,” which is characterized by mild-to-moderate impairments in memory, attention, executive functioning, and processing speed (90). The term itself and the affected neuropsychological domains resemble the “brain fog” currently used to describe NeuroPASC cognitive impairment. Furthermore, accumulating evidence suggests that chemotherapies and cranial radio-irradiation elicit a persistent microglial activation beyond the duration of treatment, leading to neuroinflammation, loss of hippocampal neurogenesis, and neuronal plasticity in addition to white matter pathology, all of which represent the core features of NeuroPASC pathology (91). Hence, it is plausible that microglial activation persists beyond the initial inflammatory stimuli in NeuroPASC, aligning with the findings observed in traumatic brain injury (TBI) and cancer-related cognitive impairment.

Globally, the resolution of neuroinflammation is essential to mitigate neurological damage. Accordingly, this is the precise role of microglia and astrocytes subsets with tissue repair and anti-inflammatory functions (33, 92). However, in neurodegenerative conditions, neuroinflammation is a crucial pathological driver as it tends to be chronically active and fails to resolve (92). Moreover, the anti-inflammatory phenotypes of microglia, which promote the clearance of inflammation in a healthy setting, are altered in neurodegenerative diseases (92). Comprehension of the delicate balance in glial cell networks and function is therefore essential to understand the complex processes governing neurodegeneration. For example, while M1 and M2 microglia are portrayed as oversimplifications to illustrate antagonistic states in both healthy and diseased brains, studies have reported distinct microglial sub-populations known as disease-associated microglia (DAM) in Alzheimer's disease (AD) (93). This unique subset of microglial cells has been specifically associated with neurodegenerative disorders and remains undetectable in healthy human brain samples. Similarly, distinct microglia populations with unique signatures have been identified in mice models, characterized by altered homeostatic gene expression and chemokine profiles that show significant overlap with DAM (41). Although the complete elucidation of DAM cells and their role in neurological disorders remains under investigation, further studies are required to map the intricate networks and function of glial cells in NeuroPASC.

## 4. Discussion

**Figure 2:**
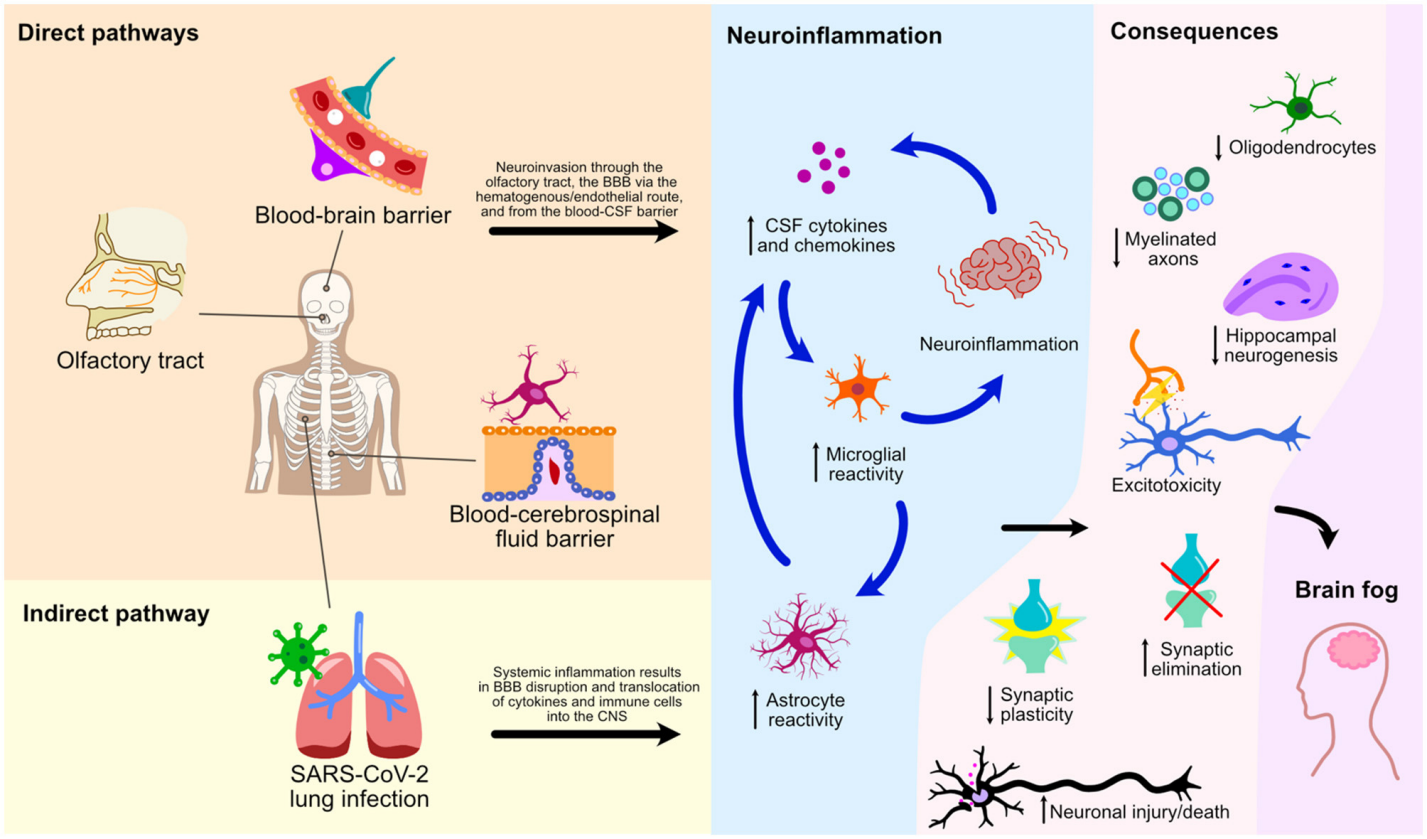
Direct and indirect pathways of NeuroPASC. Schematic representation of various pathways and their consequences on microglial reactivity and neuroinflammation of the CNS following the SARS-CoV-2 infection are depicted.

This mini-review has explored numerous cellular processes and pathways by which SARS-CoV-2 affects the CNS leading to glial reactivity and NeuroPASC (Figure 2). We illustrate an indirect pathway, characterized by the absence of direct viral invasion of the CNS, where microglial activation and neuroinflammation are consequential repercussions of systemic inflammation and BBB breakdown. These events, therefore, result in the translocation of peripheral cytokines and immune cells to the CNS, culminating in microglial activation and neurological damage. Of note, the S1 spike protein subunit of the SARS-CoV-2 could also lead to microglial priming, setting the tone for microglial reactivity and neuroinflammatory response in a viral neuroinvasion-independent manner. We herein discussed three pathways of direct neuroinvasion that could potentially lead to microglial reactivity: i) through the olfactory bulb; ii) via a hematogenous/endothelial path; and iii) through the CSF. It is likely that microglial reactivity results from a combination of these mechanisms as they are not mutually exclusive (Figure 2).

Reactive microglia are responsible for a plethora of CNS repercussions, including synaptic plasticity impairment (94, 95), inappropriate synaptic elimination, dysfunction of hippocampal neurogenesis, and memory loss (96). Secretion of the microglial pro-inflammatory cytokines also leads to numerous neuropsychiatric manifestations, including apathy, cognitive impairment, anxiety, depression, and learning disability (97). The impact of reactive gliosis has also been well documented using SARS-CoV-2 experimental models (41, 49, 52). In sum, recent data suggest that persistent neuroinflammation could explain the significant prevalence of neuropsychiatric symptoms observed in COVID-19 patients.

While controversy surrounds the legitimacy of NeuroPASC as a distinct neuroinflammatory syndrome, evidence suggests that it possesses distinct microglial subtypes (41), humoral immunity signatures (78), and T-cell activation and effector signatures (77). Despite arising from different CNS insults, the consequences of microglial reactivity, such as white matter injury, impaired hippocampal neurogenesis, and loss of myelin plasticity are similar across various syndromes. These include NeuroPASC, cancer-related cognitive impairment, cognitive dysfunction following traumatic brain injury, and influenza infection. Consequently, it should come as no surprise that the clinical translation of these shared pathological lesions takes nearly identical forms. Pharmacologically targeting these reactive pathways may hold the key to treating numerous neurodegenerative and chronic neuroinflammatory diseases.

A thorough understanding of NeuroPASC pathophysiology and microglial reactivity is primordial to the development of disease-altering therapy. It is the first step toward alleviating the important socioeconomic burden of post-acute COVID-19 syndrome and its neurocognitive sequelae, a global health problem (98).

## Author contributions

JS: literature review, original and final manuscript redaction, and review. DC: original manuscript review. GR: final manuscript review and redaction. LC-W: original and final manuscript review. All authors contributed to the article and approved the submitted version.
